# Natural Herbal Estrogen-Mimetics (Phytoestrogens) Promote the Differentiation of Fallopian Tube Epithelium into Multi-Ciliated Cells via Estrogen Receptor Beta

**DOI:** 10.3390/molecules26030722

**Published:** 2021-01-30

**Authors:** Maobi Zhu, Sen Takeda, Tomohiko Iwano

**Affiliations:** Department of Anatomy and Cell Biology, Graduate School of Medicine, University of Yamanashi, 1110 Shimo-Kateau, Chuo, Yamanashi 409-3898, Japan; mzhu-as@yamanashi.ac.jp (M.Z.); stakeda@yamanashi.ac.jp (S.T.)

**Keywords:** fallopian tube, phytoestrogen, ciliated cell, secretory cell, Notch

## Abstract

Phytoestrogens are herbal polyphenolic compounds that exert various estrogen-like effects in animals and can be taken in easily from a foodstuff in daily life. The fallopian tube lumen, where transportation of the oocyte occurs, is lined with secretory cells and multi-ciliated epithelial cells. Recently, we showed that estrogen induces multi-ciliogenesis in the porcine fallopian tube epithelial cells (FTECs) through the activation of the estrogen receptor beta (ERβ) pathway and simultaneous inhibition of the Notch pathway. Thus, ingested phytoestrogens may induce FTEC ciliogenesis and thereby affect the fecundity. To address this issue, we added isoflavones (genistein, daidzein, or glycitin) and coumestan (coumestrol) to primary culture FTECs under air–liquid interface conditions and assessed the effects of each compound. All phytoestrogens except glycitin induced multi-ciliated cell differentiation, which followed Notch signal downregulation. On the contrary, the differentiation of secretory cells decreased slightly. Furthermore, genistein and daidzein had a slight effect on the proportion of proliferating cells exhibited by Ki67 expression. Ciliated-cell differentiation is inhibited by the ERβ antagonist, PHTPP. Thus, this study suggests that phytoestrogens can improve the fallopian tube epithelial sheet homeostasis by facilitating the genesis of multi-ciliated cells and this effect depends on the ERβ-mediated pathway.

## 1. Introduction

The fallopian tube (FT) shares a developmental origin (Müllarian duct) with the uterus and serves as a route for bidirectional gamete transportation. The FT luminal wall is composed of secretory cells, multi-ciliated cells, and basal cells. Basal cells are a type of multipotent stem cell that gives rise to each specific cell type in the epithelium [1]. Secretory cells secrete mostly mucus materials that contain a series of compounds such as glycoproteins and growth factors [2]. Multi-ciliated cells have motile cilia on the apical surface to facilitate the flow of the mucous fluid [3]. Therefore, the FT cytohistological architecture is functionally adapted to cope with the physiology of the reproductive tract in vivo.

To modulate the FT environment, two major steroids, estrogen and progesterone, play important roles [4]. Previous studies suggested that estrogen can facilitate glycoprotein expression and secretion by secretory cells [2,5] and ciliogenesis in multi-ciliated cells [6], and the molecular mechanisms underlying this regulation remain unknown. Estrogen triggers the downstream pathway through the activation of estrogen receptors (ERs). Authentic estrogen receptors, ERα and ERβ, translocate from the cytoplasm to the nucleus when stimulated by estrogen, and they act as transcription factors to regulate downstream gene expression [7]. They are known to have specific targets depending on the coactivators. Knockout mouse studies showed that ERα is required for fertilization and embryonic development, and ERβ is essential for efficient ovulation [8]. However, the specific roles of ERs in conditioning the FT epithelial sheet remains unclear. Our previous study demonstrated that ERβ, but not ERα, promotes multi-ciliogenesis of FT epithelial cells by downregulating the Notch pathway [9].

Additionally, natural herbal hormone-like compounds affect the homeostasis of female reproductive tissues as an endocrine disruptor. Because all living things necessarily liaise with the external environment, they would be easily affected by ingested foodstuff. Phytoestrogen is an organic compound with estrogen-mimetic effects, and it is produced and stored in some plants such as soybeans, sprouts, and seeds [10]. Taking advantage of their effects, these plants are prescribed as unauthorized medicinal natural supplements to treat some diseases [10,11]. Conversely, a higher dosage of phytoestrogens has been suggested to stimulate the proliferation of ER-dependent breast cancer cells [12]. Among phytoestrogens, genistein, daidzein, and glycitin are categorized as isoflavones and coumestrol is a coumestan, and all of these are contained in beans and sprouts [13]. Previous studies have shown that they have a relatively higher affinity for ERβ rather than ERα [14,15,16]. Although several studies reported the effects of phytoestrogen on cancer cells [17,18,19], their actual roles in epithelial cell differentiation in the reproductive system remain unknown.

In this study, we examined the effects of phytoestrogens on the porcine FT epithelial cell differentiation using the air–liquid interface (ALI) culture system that we previously established [20]. Immunostaining of treated cells showed that phytoestrogens were able to preferentially promote multi-ciliated cell differentiation over that of secretory cells. This correlated well with downregulation of Delta-like protein 1 (DLL1), a Notch ligand, and reduced cleavage of Notch. In conclusion, the present study suggests the potential application of phytoestrogens to modulate the luminal homeostasis of the oviduct by promoting multi-ciliated epithelial cell differentiation.

## 2. Results

### 2.1. Phytoestrogens Promote Multi-Ciliated Cell Differentiation

**Figure 1:**
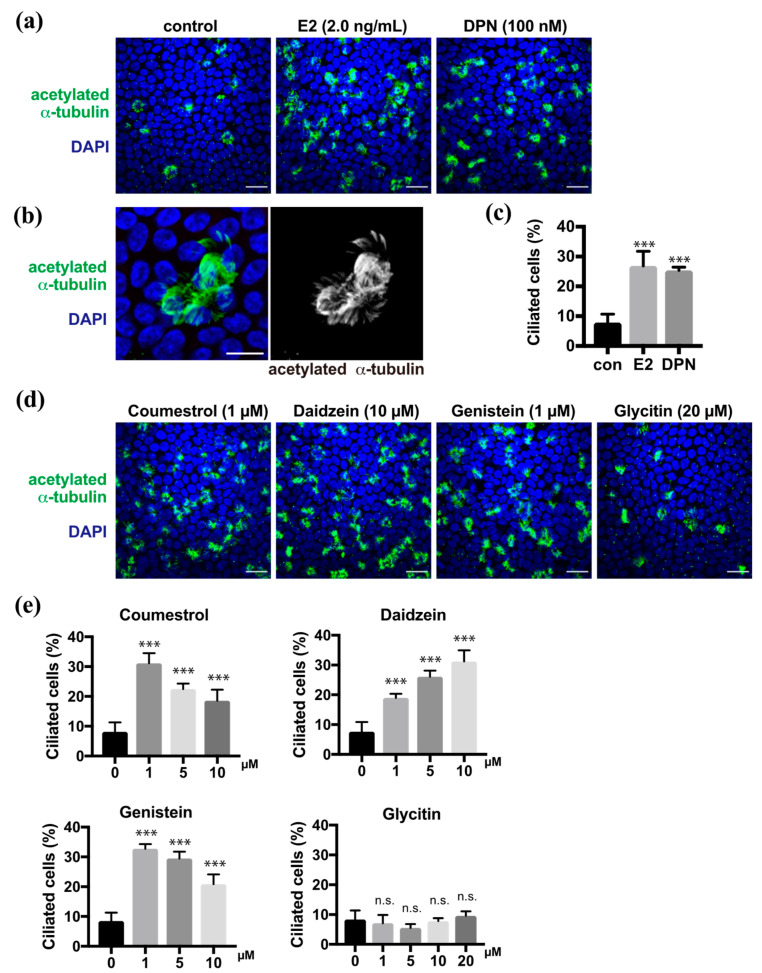
Coumestrol, daidzein, and genistein induce ciliated cell differentiation in fallopian tube epithelial cells (FTECs). (**a**) Staining for acetylated α-tubulin (green) and nuclei (blue) in porcine FTECs that were cultured for 10 days under air–liquid interface (ALI) conditions with E2 or diarylpropionitrile (DPN). (**b**) Magnified view of typical ac-tubulin-positive ciliated cells with multiple cilia. (**c**) The proportion of ciliated cells under each condition in (**a**) is presented. Data are presented as the mean ± SD (*n* = 5 fields). (**d**) Staining for acetylated α-tubulin (green) and nuclei counterstained with DAPI (blue) of cells cultured in the medium containing coumestrol, daidzein, genistein, or glycitin. (**e**) The proportion of ciliated cells under each condition in (**d**) is presented. Data are presented as the mean ± SD (*n* = 5 fields). n.s.: not significant; SD, standard deviation; DPN, diarylpropionitrile; con, control. Scale bars: 20 µm in (**a**) and (**d**); 10 µm in (**b**). Statistical significance was assigned as *** *p* < 0.001.

In the previous study, we demonstrated that estrogen promoted multi-ciliated cell differentiation through ERβ [9]. Estrogen (estradiol, E2) and diarylpropionitrile (DPN), which is an ERβ agonist, promoted the multi-ciliogenesis in approximately 30% of FTECs in the ALI culture for 10 days as shown using anti-acetylated α-tubulin antibody, whereas the negative control showed ciliogenesis in less than 10% of the cells (Figure 1a–c). To evaluate the effect of the phytoestrogens on the differentiation of FTECs, we administered coumestrol, daidzein, genistein, or glycitin in the medium (Supplementary Figure S1). All, with the exception of glycitin, promoted the multi-ciliogenesis (Figure 1d). The efficiency of coumestrol, daidzein, and genistein for inducing multi-ciliogenesis was almost 30%, which is comparable with that of E2 and DPN. Dose dependency was observed between 1 and 10 µM, where each compound showed differential dose dependency (Figure 1e). Coumestrol shared a similar pattern with genistein and showed an inverse proportion of multi-ciliated cells by increasing the concentration from 1 to 10 μM. Conversely, daidzein increased the number of multi-ciliated cells in a concentration-dependent manner. Glycitin showed no significant induction of multi-ciliated cells at any concentration. Consistently, the upregulated phosphorylation of ERβ, which is related to its activation, was observed in the nuclei of cells treated with DPN, coumestrol, daidzein, and genistein, but less with glycitin (Supplementary Figure S2). Therefore, phytoestrogens, with the exception of glycitin, can promote ciliated cell differentiation, which is similar to the cases of E2 and DPN.

### 2.2. Phytoestrogens Slightly Affect the Proportion of Secretory Cells and Basal Cells

**Figure 2:**
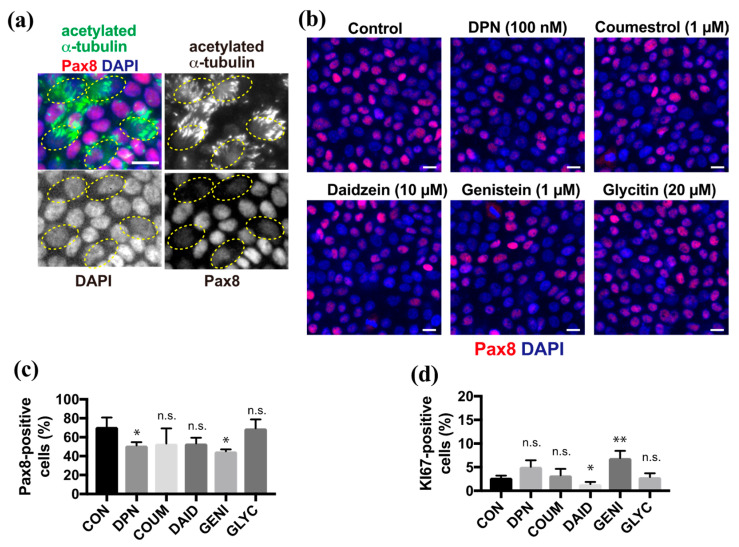
Genistein affects secretory cell differentiation and basal cell maintenance. (**a**) Staining for acetylated α-tubulin (green), Pax8 (red), and nuclei (blue) in FTECs cultured for 10 days under ALI conditions. Yellow circles indicate the ac-tubulin-positive ciliated cells with a lower Pax8 expression. (**b**) Staining for Pax8 (red) and nuclei (blue) of cells cultured in the medium containing DPN, coumestrol, daidzein, genistein, or glycitin. (**c**) The proportion of Pax8-positive in cells cultured under each condition in (**b**) is presented. Data are presented as the mean ± SD (*n* = 3 fields). (**d**) The proportion of Ki67-positive in cells that were cultured under each condition in (**b**) is presented. Data are presented as the mean ± SD (*n* = 3 fields). COUM, coumestrol; DAID, daidzein; GENI, genistein; GLYC, glycitin. n.s.: not significant; SD, standard deviation. Scale bars: 10 µm in (**b**). Statistical significance was assigned as * *p* < 0.05 and ** *p* < 0.01.

Because phytoestrogens promote differentiation into ciliated cells, we next focused on secretory cell differentiation. Pax8 is a lineage-specific transcription factor that is expressed in FT secretory cells. Multi-ciliated cells that were revealed by anti-acetylated α-tubulin antibody showed no or background levels of Pax8 in ALI culture at 10 days after induction (DAI) (Figure 2a). Upon treatment with DPN or phytoestrogens, the proportion of Pax8-positive secretory cells in the epithelial sheet varied depending on the species of compounds (Figure 2b). Coumestrol, daidzein, and glycitin did not show any significant changes in the proportion of the secretory cells compared with the control, whereas DPN and genistein slightly reduced the proportion of secretory cells compared with controls (Figure 2c). Finally, we examined the effects of phytoestrogens on basal cells because they are still present, and they replenish the epithelial sheet with differentiated cells in vivo. The proportion of basal cells that were positive for Ki67, which is a proliferation marker, did not change significantly in the presence of DPN, coumestrol, or glycitin, whereas this proportion significantly decreased and increased in response to daidzein and genistein, respectively, compared with controls (Figure 2d). Thus, several compounds affected the cell fate of secretory cells from basal cells.

### 2.3. Induction of Multi-Ciliated Cell Differentiation Depends on the ERβ Pathway

**Figure 3:**
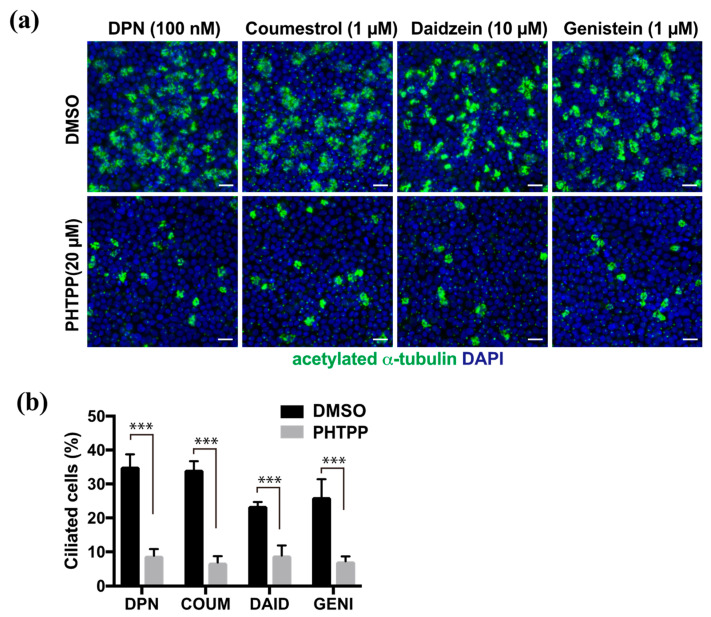
The induction of ciliated cells by phytoestrogens is suppressed by PHTPP, an ERβ antagonist. (**a**) Staining for acetylated α-tubulin (green) and nuclei (blue) of the FTECs that were cultured for 7 days in medium containing DPN, coumestrol, daidzein, and genistein with DMSO or PHTPP. (**b**) The proportion of ciliated cells that were cultured under each condition in (**a**) is presented. Data are presented as the mean ± SD (*n* = 5 fields). COUM, coumestrol; DAID, daidzein; GENI, genistein; GLYC, glycitin. SD, standard deviation; DPN, diarylpropionitrile; DMSO, dimethyl sulfoxide. Statistical significance was assigned as *** *p* < 0.001.

We previously reported that E2 promoted multi-ciliated cell differentiation through ERβ. To analyze whether phytoestrogens share this molecular pathway, we examined their effect in the presence of the ERβ antagonist, PHTPP. FTECs under ALI-culture conditions at 7 DAI were treated with DPN, coumestrol, daidzein, or genistein at their optimal concentration to induce ciliogenesis (Figure 3a). In all cases, co-administration of PHTPP significantly inhibited ciliogenesis whereas the control group without PHTPP continued to undergo ciliogenesis (Figure 3b). This indicates that ERβ is involved in the mechanism of action for phytoestrogens.

### 2.4. Coumestrol, Daidzein, and Genistein Suppress Notch Signaling

**Figure 4:**
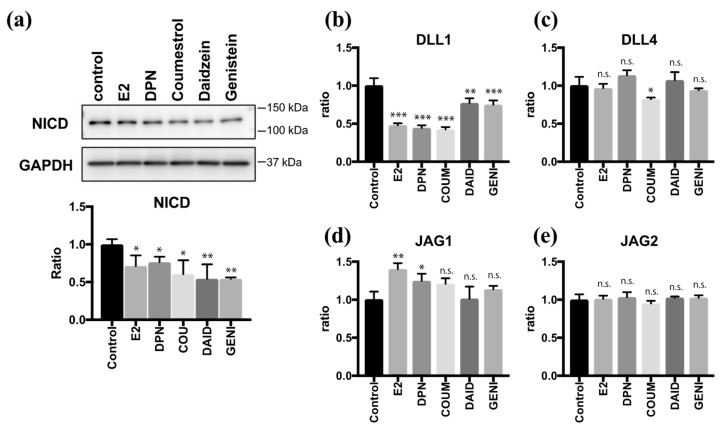
Phytoestrogens downregulate Notch signaling via suppression of DLL1. (**a**) Immunoblots for Notch intracellular domain (NICD) and glyceraldehyde 3-phosphate dehydrogenase (GAPDH) in lysates of cells that were treated for 24 h with E2 (2 ng/mL), DPN (100 nM), coumestrol (1 µM), daidzein (10 µM), and genistein (1 µM). The graph below the NICD blot shows the ratios of intensities of NICD to the GAPDH blots. (**b**)–(**e**) Quantitative RT-PCR for DLL1 (**b**), DLL4 (**c**), JAG1 (**d**), and JAG2 (**e**) expression in cells that were cultured for 24 h under each condition in (**a**) are presented. Data are presented as the mean ± SD. (*n* = 3 experiments). COUM, coumestrol; DAID, daidzein; GENI, genistein; GLYC, glycitin. SD, standard deviation; NICD, Notch intracellular domain; GAPDH, glyceraldehyde 3-phosphate dehydrogenase; DPN, diarylpropionitrile; RT-PCR, real-time polymerase chain reaction. Statistical significance was assigned as * p < 0.05, ** p < 0.01, and *** p < 0.001.

As we and another group have previously reported [9,21], the Notch signaling pathway plays an important role in regulating multi-ciliogenesis in the FT. Moreover, E2 and DPN antagonize the effect of Notch during multi-ciliogenesis. To examine the effects of phytoestrogens on Notch signaling, we quantified the level of Notch intracellular domain (NICD), which is a molecular marker of the activated Notch pathway. Twenty-four hours after treatment with coumestrol, daidzein, or genistein, the NICD level was reduced to half of the control value. This effect was attenuated by PHTPP treatment, confirming the involvement of ERβ pathway to suppress Notch signaling (Supplementary Figure S3). We further investigated the expression of Notch ligands 24 h after treatment with phytoestrogens (Figure 4a). Coumestrol, daidzein, and genistein significantly suppressed DLL1 mRNA expression but had little effect on Delta-like protein 4 (DLL4), Jagged 1 (JAG1), or Jagged 2 (JAG2) mRNA expression, and the results were consistent with those of E2 and DPN (Figure 4b–e). These data indicate that coumestrol, daidzein, and genistein suppress Notch signaling by reducing DLL1 mRNA levels during the ciliogenesis. Thus, phytoestrogens can mimic the roles of endogenous estrogen in FTEC differentiation.

## 3. Discussion

This study examined the effects of phytoestrogens on the differentiation of FTECs using the ALI culture system. We used coumestrol, daidzein, genistein, and glycitin as phytoestrogens that are included in daily foodstuff. Coumestrol, daidzein, and genistein showed significant effects in promoting multi-ciliated cell differentiation, and the effects are consistent with those of the ERβ agonist DPN. Although the differentiation of secretory and basal cells was still affected, the level was not comparable to that in multi-ciliated cells. Therefore, it is suggested that some phytoestrogens, at least coumestrol, daidzein, and genistein, act as a switch to induce ciliated cell differentiation via the ERβ pathway. However, genistein slightly but significantly reduces secretory-cell differentiation and increases Ki67-positive proliferative cells. It is possible that the promotion of ciliated-cell differentiation indirectly affects the proportion of secretory cells and basal cells. Based on the higher specificity of genistein for ERβ over ERα, the strength of fate control might depend on the ERβ pathway. Future transcriptome and epigenome analyses will reveal the downstream genes regulated by each phytoestrogen. The promotion of ciliated cell differentiation is consistent with the higher binding affinity of coumestrol, genistein, and daidzein to ERβ rather than ERα [14]. Because ciliated-cell differentiation activity was suppressed by the ERβ antagonist, the responsible pathways that promote cell fate determination may be common among the phytoestrogens. Regarding the differentiation of secretory cells, other factors or pathways might be assumed instead of the ERβ pathway, because secretory cells differentiated in about 70% of cells in basal medium alone (Figure 2b,c control).

Although we used phytoestrogens at a concentration of approximately the IC_50_ (half maximal inhibitory concentration) for each compound, this value (1–10 µM = 254–2542 ng/mL of daidzein and 270–2702 ng/mL of genistein) is much higher than the concentration (<100 ng/mL of daidzein and <160 ng/mL of genistein) in a UK cohort over 40 years of age [22]. However, the concentration of these compounds in a Japanese cohort was 0–2407 ng/mL and 0–4192 ng/mL for daidzein and genistein, respectively [22]. Therefore, the phytoestrogen concentration in our study was comparable to that in the Japanese cohort. Additionally, a US population did not show the presence of coumestrol in serum, although the urine was positive for coumestrol [23]. Therefore, we cannot assess the concentration of coumestrol.

Among phytoestrogens, our study showed that glycitin did not induce the multi-ciliogenesis. Similar to genistein and daidzein, glycitin is present in soybeans, but its structure is different from that of genistein and daidzein in terms of conjugation of glucose, and the amount of glycitin is much lower than that of other phytoestrogens [13] (Supplementary Figure S1). It has been reported that glycitin increases the ratio of Ki67-positivity in the human dermal fibroblast cells [24], although it did not induce cell proliferation in the current study. Taking the amount of glycitin in soybeans and our results into consideration, the effects of glycitin in FT epithelium would be minimal.

There are several studies that have shown different effects of genistein in different animals, such as the metabolic and secretion-stimulating action on cells, the beneficial influence on uterine homeostasis and embryonic development, and the adverse effect on implantation [24,25]. Another in vivo study in mice demonstrated that genistein reduced the efficiency of implantation [26]. Considering the very complex steps in the process of implantation, which is susceptible to various factors, it is unclear whether the adverse effect in the implantation of a mouse fertilized egg is attributable directly to genistein. Because the effect of phytoestrogens depends on the dosage and model animals that are used in each study, it is best to evaluate their effect in human cells. Although the cytoarchitecture and size of the porcine FT resembles that of humans, further elucidation of the molecular mechanism and application of phytoestrogens is required in human FTECs using primary culture system.

## 4. Materials and Methods

### 4.1. Fallopian Tube Epithelial Cell Culture

The procedures for in vitro culture and differentiation of primary porcine FTECs differentiation were described previously [20]. Briefly, porcine FT tissues were purchased from the Yamanashi Meat Logistics Center. FTECs were obtained by digesting and scraping the inside of opened porcine FTs using collagenase type IV (CLS4, Worthington, NJ, USA) and DNase I (9003-98-9, Sigma-Aldrich, St. Louis, MO, USA). To induce differentiation, cells were seeded onto a collagen type I coated 0.4-µm pore transwell (#3470, Corning, NY, USA) and basal media that included agonists or phytoestrogens were applied to the basal side in ALI culture. Reagents were as follows: β-estradiol (#E4389, Sigma-Aldrich); diarylpropionitrile (DPN) (#1428-67-7, Sigma-Aldrich); and PHTPP (#805239-56-9. Sigma-Aldrich). Phytoestrogens including genistein (#446-72-0, Wako, Osaka, Japan), daidzein (#486-66-8, Wako), glycitin (#40246-10-4, Wako), and coumestrol (#479-13-0, Cayman, Ann Arbor, MI, USA) were dissolved in dimethylformamide.

### 4.2. Immunofluorescence

The cells on the transwell were fixed using 4% paraformaldehyde (PFA) at room temperature (RT) for 10 min, permeabilized, and blocked with 0.1% Triton-X 100 and 5% goat serum in Phosphate-buffered saline (PBS) for 30 min at RT. Cells were incubated with primary antibodies (Supplementary Table S1) at 4 °C for 24 h and with secondary antibodies at RT for 1 h. Nuclei were counterstained with DAPI (D1306, ThermoFisher Scientific, Waltham, MA, USA). Transwell membranes with cells were mounted onto glass slides using Diamond Antifade Mountant (P36961, ThermoFisher Scientific). Staining images were taken using a confocal (Olympus FV-1000, Tokyo, Japan) or fluorescent (Olympus IX71) microscopes.

### 4.3. Immunoblot Analysis

Cells were lysed using radioimmunoprecipitation assay (RIPA) buffer (25 mM Tris-HCl pH 7.6, 150 mM NaCl, 1% NP-40, 1% sodium deoxycholate, 0.1% SDS) with a protease inhibitor cocktail (#04693159001, Roche, Basel, Switzerland) and phosphatase inhibitor cocktail (#07575-61, Nacalai Tesque, Kyoto, Japan). Proteins in the cell lysates were separated by a 4–20% gradient polyacrylamide gel and wet-transferred to a polyvinylidene difluoride (PVDF) membrane. The membrane was blocked using Tris-buffered saline with Tween 20 containing 2% bovine serum albumin and incubated with primary antibodies (Supplementary Table S1) at 4 °C for 24 h and with secondary antibodies for 1 h at RT. Signals were developed using an enhanced chemiluminescence substrate (#02230, Nacalai Tesque), and blotting images were acquired using an ImageQuant LAS 4000 (GE Healthcare, Chicago, IL, USA).

### 4.4. Quantitative PCR

Total RNA was extracted from FTECs using the RNeasy Mini Kit (#74104, Qiagen, Germantown, MD, USA). Double-stranded cDNA was synthesized using a reverse transcription kit (#4368813, ThermoFisher Scientific). Real-time quantitative polymerase chain reaction (RT-PCR) was performed by the FastStart Universal Probe Master kit (#04913957001, Roche) with Roche Universal Probe #2 and Probe #30 (#04684982001 and #04687639001, Roche) using a StepOne PCR system (Applied Biosystems, Foster, CA, USA). Relative RNA quantitation was performed using ∆∆CT calculations. Primer sequences are listed in Supplementary Table S2.

### 4.5. Statistical Analysis

Statistical analyses were performed using GraphPad Prism 7 (San Diego, CA, USA). Values were expressed as the mean ± standard deviation (SD). The Student’s t-test was used to compare the variation between two samples. Analysis of variance (ANOVA) was used to compare three or more groups. Statistical significance was assigned as * *p* < 0.05, ** *p* < 0.01, and *** *p* < 0.001.

## Data Availability

The data that support the findings of this study are available from the corresponding author upon reasonable request.

## Supplementary Materials

The following are available online, Figure S1: Molecular structure of phytoestrogens that are used in this study; Figure S2: Phytoestrogens activate ERβ.; Figure S3: Phytoestrogens downregulate Notch signaling via ERβ pathway.; Table S1: List of antibodies used in this study; Table S2: List of oligonucleotides used in this study.
