# Supplementary material for: Natural Herbal Estrogen-Mimetics (Phytoestrogens) Promote the Differentiation of Fallopian Tube Epithelium into Multi-Ciliated Cells via Estrogen Receptor Beta
Source: Molecules. 2021 Jan 30;26(3):722. doi: 10.3390/molecules26030722 (PMC7866512; doi:10.3390/molecules26030722)
Supplement: Supplementary file 1 [file molecules-26-00722-s001.pdf]

Supplementary figure S1

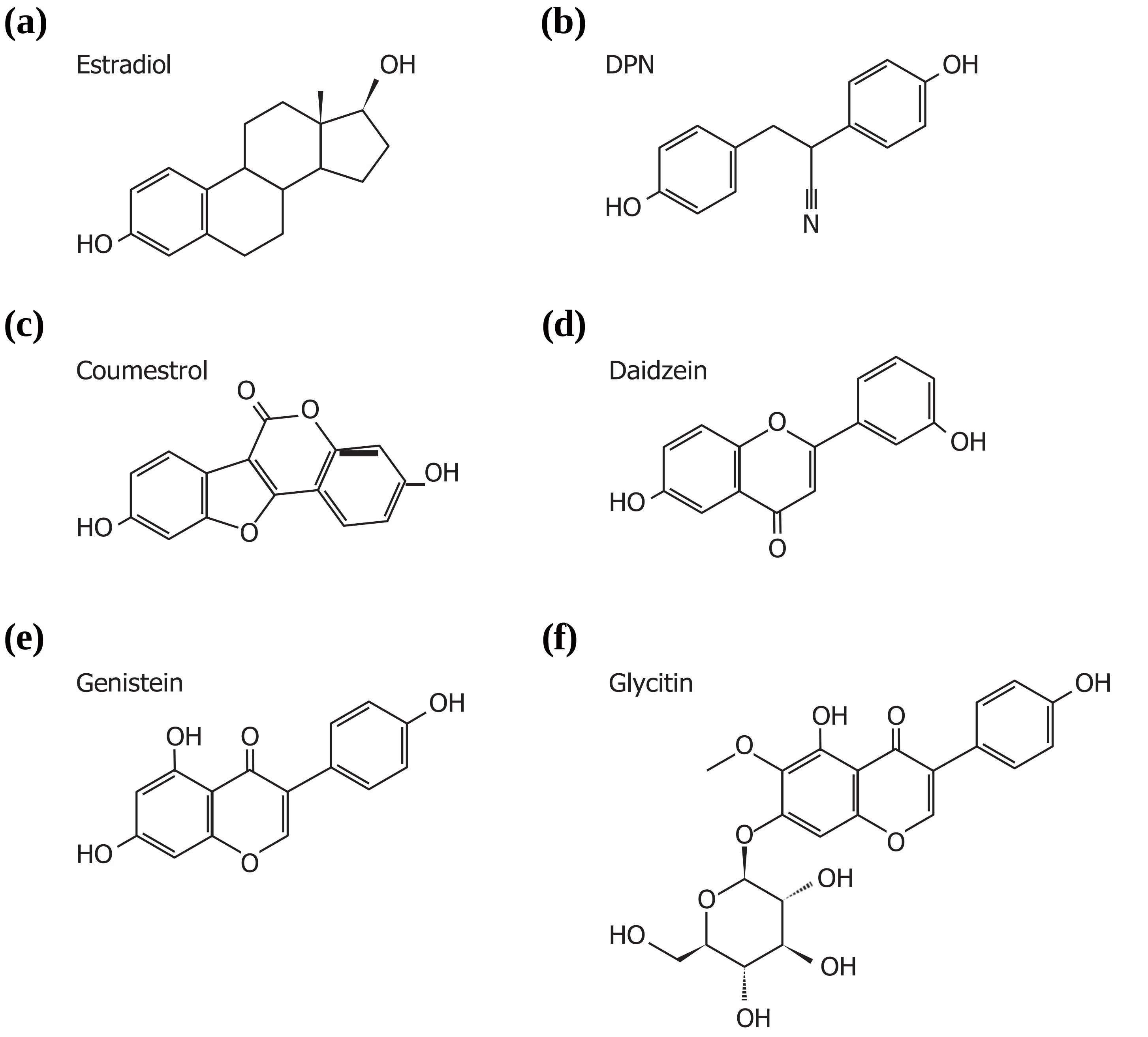

Supplementary figure S1 Moreluclar structure of phytoestrogens that are used in this study. (a) Estradiol (E2); (b) DPN, diarylpropionitrile; (c) coumestrol;(d) daidzein; (e) genistein; (f) glycitin.

Supplementary figure S2

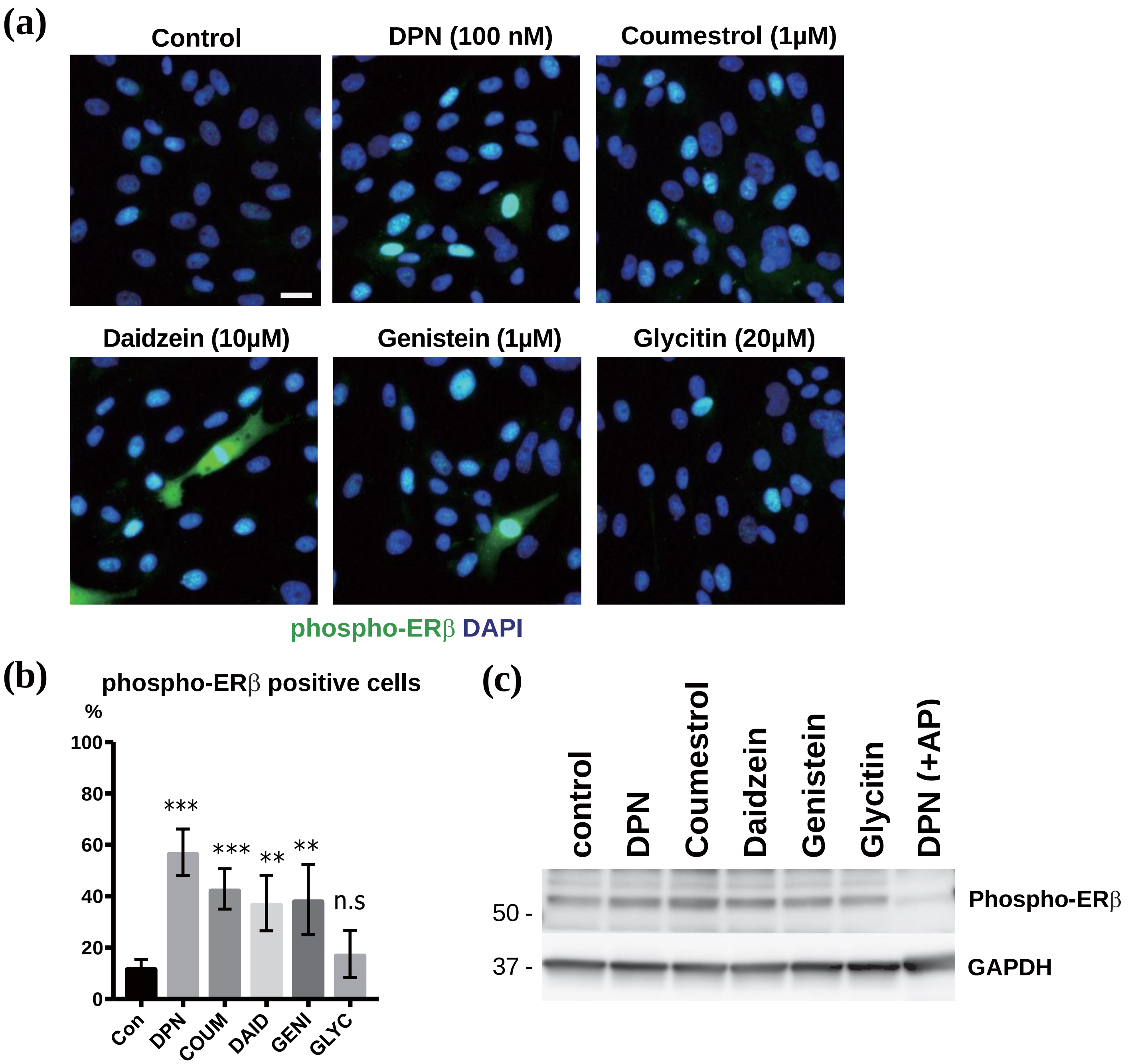

Supplementary figure S2 Phytoestrogens activate ERβ. (a) The FTECs that were cultured for 24h in the medium containing DPN (100 nM), coumestrol (1 μM), daidzein (10 μM), and genistein (1 μM) or glycitin (20 μM). To analyze the activation of ERβ, phosphorylated (phospho-) ERβ was immunostained by specific antibody (green) and nucleus was counter-stained with DAPI (blue). (b) The proportion of cells in which phospho-ERβ is highly expressed in the nucleus is summarized in the graph. Data are presented as the mean ± SD (n=5 fields). (c) Immunoblots for phospho-ERβ and GAPDH are shown for the cells that were treated for 24h with DPN, coumestrol, daidzein, genistein or glycitin. The signals of phospho-ERβ in the treatment of DPN, coumestrol, daidzein and genistein were slightly increased but not obvious because of the high basal level in bulk protein samples. As a negative control, DPN-treated cell lysate was incubated with AP. This band (the most right lane) was very weak , indicating the antibody specificity for phosphorylation. DPN, diarylpropionitrile; COUM, coumestrol; DAID, daidzein; GENI, genistein; GLYC, glycitin. SD, standard deviation; n.s., not significant, AP. alkaline phosphatase.

Supplementary Table S1

List of antibody used in this study

name	host	cat. number	Company	State, Country
anti-acetylated α-tubulin	mouse	6-11B-1	Sigma	MO, USA
anti-Pax8	rabbit	10336-1-AP	Proteintech	IL, USA
anti-Ki67	rabbit	NCK-Ki67-P	Leica	Wetzlar, Germany
anti-GAPDH	mouse	60004-1-Ig	Proteintech	IL, USA
rabbit anti-Notch1	rabbit	ab52627	Abcam	Cambridge, UK
HRP-conjugated anti-mouse IgG	goat	7076	CST	CO, USA
HRP-conjugated anti-rabbit IgG	goat	7074	CST	CO, USA
Alexa Fluor 488 conjugated anti-mouse IgG	donkey	A21202	ThermoFischer scientific	CA, USA
Alexa Fluor 568 conjugated anti-rabbit IgG	donkey	A10042	ThermoFischer scientific	CA, USA

Supplementary Table S2

List of oligonucleotide used in this study

Gene name	orientation	Sequence
DLL1	Sense	5’-GAATGGAGGGAGCTGCAC-3’
	Antisense	5’-CACTCACGCAGATCCT-3’
DLL4	Sense	5’-GTGGTGCTGGTGGTACTGTG-3’
	Antisense	5’-AGTCCGACAAGTTGTTCATGG-3’
JAG1	Sense	5’-CTCACAGCTATGCAAACACCA-3’
	Antisense	5’-CCTAAGACTGCATCACCATCTG-3’
JAG2	Sense	5’-GCCCAATCCCTGTGTGAA-3’
	Antisense	5’-GGTATTGTGCGTGCAGGTT-3’
ATP5F1	Sense	5’- CACGTGGTGCAGAGCATC-3’
	Antisense	5’-TCTTTGCGAGCAGCTTTAGA-3
